# Therapeutics

**Published:** 1876-02

**Authors:** 


### IV. THERAPEUTICS.

1. *Treatment of Tapeworm by Creosote.* Mr. BRICKWELL. (*Med. Times and Gaz.*, Sept., 1875.)

The writer states that about fifteen years ago a man discharged from the army with lung disease and tapeworm came under his notice, when it occurred to him that the destructive properties of creosote to the lower grades of animal life might be made available for killing or so weakening the vitality of intestinal worms that they could easily be got rid of. He therefore gave him some, three times a day, shortly after meals, for six days, and on the seventh a dose of castor-oil and turpentine, which brought away a worm twelve yards long. He has since tried creosote for destroying round worms with great success. In one case a large mass of more than a hundred worms of all sizes came away, and the patient has not been further troubled by them. He has not succeeded so well with the troublesome thread-worms of adults, but has some doubts if the remedy were fairly tried. He has just had the following case of tapeworm :—W. E., aged twenty-three, laborer, pale, sallow, and sickly-looking, but fairly fleshy, came to Mr. Brickwell with hæmoptysis and symptoms of tubercle in the lung, which passed off, and he was able to work through the winter. Four months ago he came to him suffering with the prevailing malady, bronchitis, and he then first began passing portions of tapeworm. He deferred treating him for the latter until his health was better. A month ago he returned in fair health, having frequently passed portions of worm since his last visit. Mr. Brickwell gave him one drop of creosote made into a pill, with pulv. tragacanthæ co. three times a day, half an hour after each meal, so as to impregnate the ingesta. As the stomach bore the medicine well for two days, he increased the dose to two drops three times a day for two more days, and then to three drops at a time for two more days. On the seventh day he gave him a dose of castor-oil (aperients acted strongly on the patient, or he would have combined it with sp. terebinthinæ). On the fifth day of the treatment, a worm eight yards long came away, and he had slight diarrhœa. Not seeing Mr. Brickwell, he continued the creosote on the sixth day, and took the oil on the seventh, which acted very freely, but brought no more worm away. There appear to have been no symptoms to indicate the presence of the worm until he began to pass portions of it. He had, however, complained of a feeling of weakness, sinking and faintness, for several years, more particularly in the evening. These feelings have now disappeared, and he is stronger, better, and more healthy-looking than before. The two points that struck Mr. Brickwell are—first, that he began passing the worm during a febrile attack (could that have influenced the vitality of the worm, and so tended to its passage ?) ; and, secondly, that he had a chest complication. It is a curious coincidence, that these symptoms should have co-existed in the only two cases of tapeworm which have come under his notice.

2. *On Treatment by Means of the Stomach Pump.* Dr. PAUL SCHLIEP (*Deutsche Arch. f. Klin. Med.*, B. xiii ; *The Practitioner*, 1875.)

Dr. Paul Schliep, of Berlin, has treated seventy-four cases of disease of the stomach in 982 sittings, with Küssmaul's stomach-pump, and obtained very satisfactory results. Among these were two cases of poisoning, one of arsenic and the other by a spirituous solution of lac ; both cases recovered. Twenty-five cases of simple gastric catarrh (the pump being employed on the average nine times in each case) ; seventeen cases of gastric catarrh, complicated with other conditions, of which four cases were complicated with chlorosis, five with nerve lesion, four with pulmonary disease, two with jaundice, two with neoplastic formations in other organs, ten with ulcus ventriculi (three acute cases, the pump being used in the chronic cases about fourteen times) ; fourteen cases of dilatatio ventriculi (of which five were cured and four improved, and in which the pump was applied on the average twenty-one times) ; six cases of carcinoma ventriculi. Dr. Schliep, it will be seen, thus treated conditions with the stomach catheter which are usually left alone ; and on the other hand, conditions, as ulcer and carcinoma, which it is usually considered unadvisable to treat with instruments that are likely to produce movements of vomiting.

The treatment of simple gastric catarrh with the pump is justified by Dr. Schliep, first of its success, and secondly, on its obstinacy under other methods. He found great benefit from it, even after a few sittings only—and explains it on the same principle that brilliant results are often obtained from an emetic. A recent gastric ulcer is often a *noli me tangere*, but considerable benefit was experienced in chronic and cicatrized ulcers. In cases of dilatation of the stomach, Dr. Schliep commenced his treatment with daily complete evacuation of the stomach. If the muscular coat resumes its contractility, and the atrophy and degeneration of the glandular tissue have not proceeded too far, the abnormal decomposition that occurs in the gastric contents soon disappears. The effects of this mode of treatment on cancer of the stomach were decidedly alleviating. In performing the operation the patient must attend carefully to his breathing, must not bite the tube, and must first hold the head backwards, in order that the obstacle presented by the posterior wall of the pharynx may be surmounted, and then forward, in order to convert the projection formed by the bodies of the third and fourth cervical vertebræ into a concavity. Having entered the cardia, which can be recognized by the length to which the tube has passed, and a slight resistance, an effort should be made to see whether any anatomical change has taken place, which may be known by the occurrence of pain, strong resistance to the entrance of the instrument, and perhaps blood, mingled with the contents of the stomach when they have been pumped out. He finds cases of dilatation of the œsophagus above a stricture at the cardia offer the greatest difficulty to the introduction of the sound. The sound having been introduced, M. Schliep begins with the injection of several syringesful of water at about the temperature of the body, until the patient feels the stomach to be full, or vomiting sets in, after which he carefully extracts the fluid again. Care must be exercised in securing an instrument that works easily and well, so that the wall of the stomach may not be sucked into the openings of the sound. He does not attach any importance to slight tinging of the contents with blood, and remarks that strong alkaline solutions introduced into the stomach produce general hyperæmia and consequent bloody straining of the contents.

In order to remove adherent mucus from the walls of the stomach, he recommends that the patient should be made to execute several movements of vomiting by tickling the fauces, by means of movements of the tube, though this is usually unnecessary, movements of vomiting occurring spontaneously. In other cases, and especially where an ulcer is present, vomiting should be avoided as much as possible. Considerable caution should be exercised in withdrawing the tube, lest portions of the mucous membrane which have got sucked into the holes should be torn away.

Dr. Schliep distinguishes the contents of the stomach in different states into—1. Clear mucus, with neutral reaction, met with in simple chronic catarrh ; 2. Yellow fluid, with neutral reaction, accompanying catarrhal inflammation of the stomach and duodenal mucous membrane in scleria ; 3. Mixed contents, composed of undigested food and mucus, with, for the most part, slightly acid reaction ; 4. Contents in a condition of acid fermentation, occurring in recent inflammatory conditions and ulcer ; the prognosis is here good ; the removal essentially effects a cure ; 5. Contents containing abundance of fungi (Leptothrix, oidium albicans, sarcina), reaction acid, quantity sometimes extraordinarily great ; the regular evacuation of the contents of the stomach greatly tends to remove the accompanying dilatation; 6. Putrescent contents (coffee-grounds like) often accompanying cancer of the pylorus, with dilatation of the stomach ; the quantity is here again very large and the reaction acid ; 7. Abnormal gaseous contents.

Among the remedies used, Dr. Schliep enumerates the following as having been pumped in and again evacuated : Bicarbonate of soda, of which two teaspoonsful were added to a wash-hand-basinful of water, and injected, in cases where the reaction of the contents was strongly acid; potassium permanganate in the proportion of 1 to 100 of water, the quantity injected being again a wash-hand-basinful of water; used in cases where the contents were putrescent ; carbolic acid, in the proportion of 1 to 40, though in this case the acid solution should not be allowed to remain long in contact with the mucous membrane ; chloralum, boracic acid and tincture of myrrh, were respectively used in weak solutions, when the quantity of mucus secreted was large, and the membrane torpid. The diet was carefully selected, and food given only in small quantities at a time.

3. *Camphor in Erysipelas.* (*Gaz. des Hôpiteaux*, 1874.)

M. Revillout states that he has several times had occasion to employ, with good effect in erysipelas, an application used by M. Delfech at the Neker. It consists in painting the affected surface with a solution of camphor in ether (equal weights); and when this is employed in erysipelas of the face, and the affection has not yet reached the hairy scalp, its progress is usually arrested. It is also very useful in erythema, caused by local irritation.

4. *On Surgical Anæsthesia in Infants by the Administration of Chloral by the Stomach.* BOUCHUT. (*Bulletin Gén de Thérapeutique*, Oct. 30, 1875.)

The writer gives his conclusions as regards the action of chloral in infants, which differs materially from that in adults. It may be given in doses up to forty-five or sixty grains, without provoking pyrosis, gastritis, vomiting, or diarrhœa, and it produces anæsthesia more or less complete. He has employed it repeatedly in cases of chorea, cerebral rheumatism, opening abscesses, and tooth-drawing. Sleep and absolute insensibility have been obtained by a dose of forty-five to sixty grains given in one draught.

A similar result may be obtained by means of suppositories, but the rectum is more irritable than the stomach, and tenesmus, heat and irritation occur if the method be employed for many days. The stomach is the best channel for the administration of chloral in infants. A quarter of an hour after taking the full dose anæsthesia commences, and is completed at the end of an hour.

When it is necessary to apply Vienna paste to a nevus, or to open an abscess, or draw a tooth, chloral is indicated.

The child may groan and move, without waking, whilst the operation is being performed, but then falls again into a passive condition, and on awakening some quarter of an hour later, it is unconscious of having suffered pain, and has been spared the fright of seeing the operator.

